# Isolated trochlear nerve palsy with midbrain hemorrhage

**DOI:** 10.4103/0301-4738.58476

**Published:** 2010

**Authors:** S Raghavendra, K Vasudha, S Ravi Shankar

**Affiliations:** Department of Neuroophthalmology and Department of Neuropathology, Narayana Netaralaya, Bangalore, India

**Keywords:** Isolated trochlear nerve palsy, midbrain cavernoma

## Abstract

Midbrain hemorrhage causing isolated fourth nerve palsy is extremely rare. Idiopathic, traumatic and congenital abnormalities are the most common causes of fourth nerve palsy. We report acute isolated fourth nerve palsy in an 18-year-old lady due to a midbrain hemorrhage probably due to a midbrain cavernoma. The case highlights the need for neuroimaging in selected cases of isolated trochlear nerve palsy.

Idiopathic, traumatic and congenital abnormalities are the most common causes of isolated fourth nerve palsy. Midbrain hemorrhage causing isolated fourth nerve palsy is extremely rare.[1] We report a patient with acute isolated fourth nerve palsy due to a midbrain bleed probably due to a cavernoma.

## Case Report

An 18-year-old lady presented with four weeks of binocular vertical diplopia that was accentuated in the downward gaze. She also developed tinnitus episodically in the right ear. She gave no history of any systemic disease.

On examination, her uncorrected visual acuity was 20/20, N6 in both eyes. Her head position was normal. Her extraocular movements in the right eye were normal. Her left eye showed over-action of inferior oblique (+2) and under-action of superior oblique (−2). Cover test showed a small left hypertropia that increased on left head tilt (Beilschovosky's test positive). The left hypertropia was more in the right down gaze. Diplopia charting with red green goggles (red in front of the right eye and green in front of the left eye) showed vertical diplopia, with the red image higher than the green image, in the primary position, dextroversion, on down gaze and on right down gaze. The separation of images was maximal in the right down gaze. Pupils were equal, normally reacting to light. There was no relative afferent pupillary defect. Near reflex was present. Convergence was normal. The fundus examination with indirect ophthalmoscopy was within normal limits. Slit-lamp examination showed a normal anterior segment in both eyes. Corneal sensations were normal in both eyes. Facial nerves were normal. Hearing was clinically normal. Rinne's test was positive bilaterally and Weber's equal. Throat and neck movements were normal. There were no motor, cerebellar or sensory abnormalities in the limbs.

**Figure 1:**
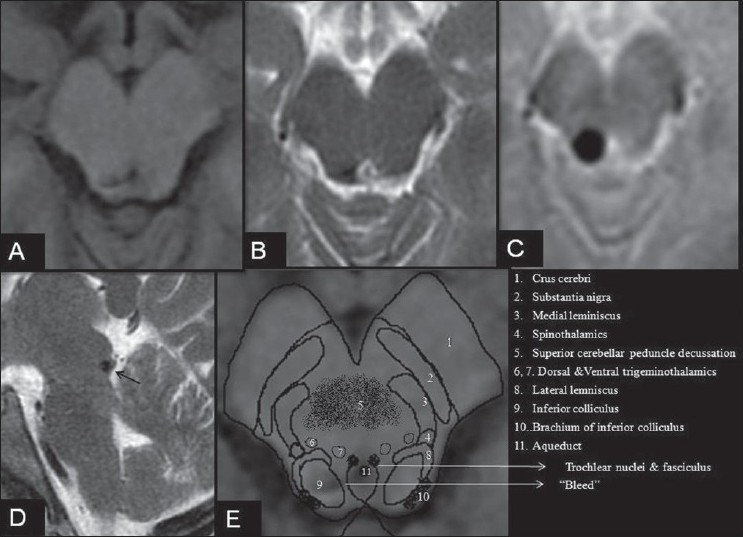
MRI brain in a patient with isolated acute left trochlear nerve palsy. Axial T1 SE (A), axial T2 FSE (B), Axial T2*GRE (C) and Sagittal T2WI(D) showing a hypointense lesion in the right tectal region at the level of the inferior colliculus with intense blooming on gradient imaging without any flow voids. E, schematic representation of the anatomical structures of the midbrain at the level of the inferior colliculus overlapped on Figure A. Note: Increased hypointensity of a paramagnetic substance such as blood on T2*GRE (gradient) imaging is termed as blooming

Magnetic resonance imaging (MRI) brain including contrast study showed a dorsal midbrain lesion in the area of right tectal plate that was hypointense on T2 weighted image and showed blooming on susceptibility and gradient T2 imaging without any flow voids surrounding the area located [Fig. 1 A–D].

She was given prisms in the lower segment of the glasses to alleviate her diplopia in down gaze. In view of the strategic location and the minimal deficit a decision for close follow-up and intervention if new symptoms develop was taken. At six months follow-up, she had no tinnitus or hearing disturbance and her deficits remained same. A repeat MRI showed the lesion to be static.

## Discussion

We here report a patient of isolated fourth nerve palsy due to a small tectal hemorrhage secondary to a probable midbrain cavernoma. Few cases of isolated trochlear nerve palsy with midbrain hemorrhage or due to midbrain vascular malformation are reported in the literature.[1–5] Sürücü *et al.* very recently reported a patient of cavernoma with symptomatic fourth nerve palsy that was surgically treated.[6]

Chen *et al*. reviewed the reported world literature of nine patients with midbrain tectal bleed and noted unique presentation of diplopia along with either contralateral tinnitus or sensory disturbance. The transient tinnitus observed in our patient was probably due to involvement of the inferior colliculus.[1]

Axons of the trochlear nerve originate from the nucleus in the ventral periaqueductal grey at the level of the inferior colliculus. These axons indent the dorsal surface of the medial longitudinal fasiculus, curve dorsolaterally and caudally to reach the superior medullary velum to decussate with the nerve of the opposite side and exit from the brainstem [Fig. 1 E]. The auditory afferents from the cochlear nucleus in the medulla oblongata after traversing the superior olivary nucleus and nucleus of lateral lemniscus in the rostral pons, pass through the inferior colliculus and adjacent lateral lemniscus in the lower midbrain. The efferents are bilateral but dominantly contralateral to the inferior colliculus. The auditory fibers from the inferior colliculus traverse the medial geniculate body to reach the cortex in the temporal lobe. The combination of ocular and auditory symptoms emphasizes the neuroanatomical contiguity of the trochlear nerve pathway and the adjacent inferior colliculus [Fig. 1 E].

In the present case, the associated audiological symptoms could be due to irritation of the auditory pathway traversing the ipsilateral inferior colliculus by the seeping blood products, altered calcium homeostasis and disinhibition of GABAergic transmission in the neurons and axons in the close vicinity.[7–9]
